# Failure Rate and Early Complications of Thumb Carpometacarpal Joint Replacement—A Multicenter Retrospective Study of Two Modern Implant Designs

**DOI:** 10.3390/jcm13010121

**Published:** 2023-12-25

**Authors:** Uri Farkash, Mojahed Sakhnini, Daniel Dreyfuss, Daniel Tordjman, Gilad Rotem, Shai Luria

**Affiliations:** 1 Hand Surgery Unit, Department of Orthopedic Surgery, Assuta-Ashdod University Hospital, Ashdod 7747629, Israel; 2 Faculty of Health Sciences, Ben Gurion University of the Negev, Beer Sheva 8499000, Israel; 3 Department of Orthopedic Surgery, Rivka Ziv Medical Center, Safed 1304435, Israel; mojahed.sakh@gmail.com; 4 Hand and Microsurgery Unit, Rambam Health Care Campus, Haifa 3525408, Israel; d_dreyfuss@rambam.health.gov.il; 5 Rappaport Faculty of Medicine, Technion, Haifa 3525408, Israel; 6 Hand Surgery Unit, Orthopedic Division, Tel-Aviv Sourasky Medical Center, Tel Aviv 6423906, Israel; dm.tordjman@gmail.com; 7 Sackler Faculty of Medicine, Tel-Aviv University, Tel Aviv 6139001, Israel; gilad.rotem@sheba.health.gov.il; 8 Department of Hand Surgery, Sheba Medical Center, Tel Hashomer 5262000, Israel; 9 Department of Orthopedic Surgery, Hadassah Medical Center, Jerusalem 9371125, Israel; shail@hadassah.org.il; 10 Faculty of Medicine, Hebrew University of Jerusalem, Jerusalem 9112102, Israel

**Keywords:** trapeziometacarpal osteoarthritis, carpometacarpal osteoarthritis, arthroplasty, prosthesis, postoperative complications

## Abstract

Joint replacement arthroplasty for the treatment of thumb osteoarthritis is gaining popularity as recent studies have demonstrated better pinch and grip strength and faster rehabilitation. Our aim was to identify early complications in modern implant designs using a multicenter study. A total of 381 patients who underwent thumb carpometacarpal replacement surgery in six participating hospitals were enrolled. The complications included were fractures, dislocations, infections, tendon and nerve injuries, and complex regional pain syndrome. Major complications were defined as a failure to implant the prosthesis, revision surgery to remove the implant, and any other need for further surgical intervention. The secondary outcomes were any other complications treated non-surgically and the timing of the complications. Eleven procedures failed, and these patients were treated with trapeziectomies. Twelve other patients required repeat surgical interventions. Minor adverse events occurred in 25.4% of the cases, and transient irritation of the superficial radial nerve and De Quervain tendinopathy were the most prevalent complications. Although this cohort depicted the learning curves of multiple surgeons, our study demonstrated low short-term failure rates. An inability to achieve primary stability of the cup in the trapezium was the leading cause of failure. Dislocations and other major complications with modern implants were very few.

## 1. Introduction

Osteoarthritis of the first carpometacarpal (CMC) joint is an extremely common disease that has an age-adjusted prevalence of 7% for men and 15% for women [1]. CMC joint osteoarthritis can cause pain, deformity, limited range of motion, joint instability, and weakness, all of which can lead to functional disability, most notably in postmenopausal women and elderly populations [2]. The overall goal of treatment is to relieve pain, improve thumb motion, provide joint stability, and improve hand function [3]. Non-surgical treatments include activity modification, oral pain-relief medication, splints, physiotherapy, and corticosteroid injections [4]. Surgical interventions are indicated when symptoms have not been controlled despite non-operative therapy; these include extension osteotomy, CMC arthroscopy with debridement, trapeziectomy alone, trapeziectomy with ligament reconstruction and tendon interposition (LRTI), trapeziectomy with suspensionplasty, arthrodesis, or joint replacement (JR) [2].

Trapeziectomies were introduced as surgical treatments for CMC osteoarthritis more than 70 years ago, with reports of generally good pain reduction and restoration of thumb mobility [5]. However, shortening of the thumb has been a concern that is believed to reduce pinch strength and produce impingement of the metacarpal base with the scaphoid. Ligament reconstruction and tendon interposition were added to reduce this complication. Over the years, alternative procedures have been sought and have produced debatable benefits.

The concept of interposition implants was introduced in an attempt to restore pinch strength by maintaining the length of the thumb and filling the space left by a trapeziectomy. The first generation of implants was comprised of silicone spacers [6]. Subsequently, de la Caffinière introduced a total joint replacement that converted the CMC saddle joint into a ball and socket joint by incorporating a cup in the trapezium and a stem in the first metacarpal [7]. Initially, this procedure involved the use of cemented prostheses. Due to high complication and revision rates, implant designs were changed over the years [8]. Cementless metal-on-polyethylene implants were introduced in the early 2000s, primarily featuring grid-blasted titanium or hydroxyapatite-coated cobalt chrome components. Subsequently, a metal-on-metal prosthesis was developed, aiming to reduce cup size for a better fit within the smaller trapezium. However, due to high complication and revision rates, the use of metal-on-metal implants was eventually discontinued. Over the last decade, attention has shifted towards enhancing cementless ball and socket implants with metal-on-polyethylene articulations. This evolution in implant design reflects a continuous effort to optimize functionality and longevity while addressing the challenges encountered with earlier prostheses designs.

Modern ball-and-socket CMC implants are cementless and use a metal-on-polyethylene friction couple and grid-blasted titanium or hydroxyapatite-coated chrome–cobalt components. There are several cup designs (hemispheric, conus, or screw) and stems on the market, as well as implants with dual-mobility articulation. 

Historically, the prevailing notion suggested that total CMC joint replacements have limited benefits compared with trapeziectomies because of the high revision rates and the suboptimal long-term implant durability. Nonetheless, recent publications have presented a shift in perspective by comparing thumb CMC joint replacement with trapeziectomies in non-randomized studies spanning over 12 months of follow-up. These studies have revealed better pinch and grip strength with JRs and faster rehabilitation [9,10]. Consequently, these findings have contributed to a surge in the adoption and favorability of thumb CMC joint replacement procedures, challenging the previously held belief regarding its limited benefits compared to a trapeziectomy.

Studies describing JR surgery complications are limited. Such studies have largely described single-surgeon experiences and previous generation implants and have analyzed relatively small sets of patients. While meta-analyses have also been performed, these are limited by the inherent limitations in the different study designs, including variable reporting of outcomes, surgical techniques, and publication biases. Our aim was to identify early complications in thumb CMC JRs in a multicenter study, with multiple surgeons and specific attention paid to the survivorship of the prostheses. Such data are valuable for surgeons in providing the percentages of risks for major and minor complications when obtaining consent from patients receiving JRs.

## 2. Materials and Methods

All patients who underwent thumb CMC JR surgeries in six participating hospitals between 1 May 2015 and 31 December 2022 were included. The study was approved by each participating hospital’s ethics committee. Data from patients’ records were retrospectively collected, including patient demographics (including age and gender), occupation, and hand dominance. The surgical data included the surgical approach and the implant type and size. All operations were performed by surgeons with level two or three expertise according to Tang et al. [11]. The follow-up data included intra- and post-operative complications diagnosed clinically or radiographically for a period of at least one year. 

The complications included were fractures of the trapezium or metacarpal bones, dislocations of the prosthesis, infections, tendon and nerve injuries, and complex regional pain syndrome. The timing of the complications in relation to the timing of the surgeries was also recorded. The primary outcome measures were major complications, defined as failures to implant the prostheses, revision surgeries to remove the implants, and any other need for further surgical intervention related to the JR surgeries. The secondary outcomes were any other minor complications treated non-surgically and their timing. 

The quantitative variables are described as means, standard deviations (SDs), and ranges (minimum–maximum). The qualitative variables are described as numbers and percentages. Chi-square tests were used to compare the categorical variables. The level of significance for all tests was set at a *p*-value of less than 0.05.

## 3. Results

In the six participating medical centers, 381 patients were treated with thumb CMC JR surgeries during the study period. The mean age of the patients was 63 ± 9 years (range of 39–84), with a female majority (298; 78%). Although 93% of patients were right-hand dominant, the surgeries were conducted nearly equally (186 right and 195 left) in both hands. In all, 37% of the patients were retired or did not work, 28% were low physical-demand workers, and 35% were high physical-demand workers. 

**Figure 1:**
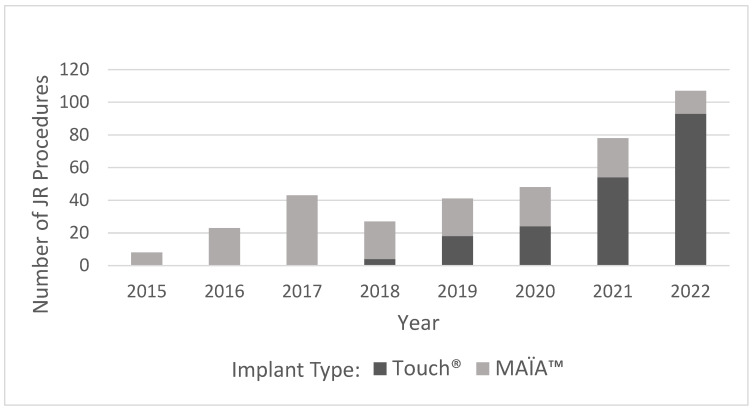
Number of JR procedures performed during the study period.

During the study period, there was an increase in the number of procedures performed as more surgeons in more hospitals began to adopt the JR technique into their practice (Figure 1). The effect of the COVID pandemic could explain the pause in the increase in the number of surgeries during 2020. The mean follow-up period was 3 ± 2 years. 

In the early years of the study, the MAÏA™ prosthesis (Groupe Lépine, Genay, France) was used. In the year 2018, the Touch^®^ prosthesis (KERIMEDICAL, Geneva, Switzerland) was introduced (Figure 1). The choice of implant and surgical approach were at the surgeon’s discretion. In 181 of the procedures, a MAÏA™ implant was used—most frequently (159 cases) the single-mobility semiretentive design—and in 19 cases, a double-mobility MAÏA™ implant was used, while there were three cases where the data were missing. The Touch^®^ implant, which has a double-mobility design, was used in 200 cases, and in 84 of them, a spheric cup was used, while in 112, a conical cup was used. In four cases, these data were missing. Most procedures (296, 78%) were completed through a dorsal approach, and the other 22% were carried out using a radiopalmar approach. 

Eleven JR procedures (2.9%) failed, and these patients were treated with trapeziectomies with or without ligament reconstruction or suspensionplasty at the surgeon’s discretion and were classified as having major complications (Table 1). In two cases, the surgeon faced difficulty stabilizing the cup in the trapezium during the initial surgery due to a fracture of the trapezium, leading to an intra-operative decision to proceed with a trapeziectomy. In nine other cases, the patients were returned to the operation rooms at later stages for trapeziectomies. Late trapeziectomies were necessitated for various reasons, including early cup-loosening (four cases, with trapezium fractures in three of them during the initial surgery), recurrent dislocations resistant to stabilization despite a switch to a longer neck (one case), fracture of the trapezium resulting from a fall on the hand (6 weeks post-initial surgery; one case), deep infection (8 months after the initial surgery; one case) and two cases of persistent pain at the base of the thumb for a year without abnormal findings on physical examination or imaging studies. 

An additional twelve patients underwent surgical intervention to address complications arising from their JR surgeries (Table 1). Three cases involving cup-loosening were managed by re-stabilizing the same cup or revising to a larger-diameter cup. Dislocation of a single-mobility implant in one case was addressed by revising to a dual-mobility design. Mechanical implant failures in two cases led to revisions of the dislocated components. Six other revision surgeries were conducted to address complications unrelated to the implants themselves.

The minor adverse events are listed in Table 1, and they resulted in an overall postoperative complication rate of 31%, with no statistical differences between the high physical-demand workers and the retired or low physical-demand workers (33% and 29%, respectively). Complications occurred in all participating hospitals and were not found to be more prevalent in certain hospitals or in certain implant designs or sizes. The complication rate was not influenced by the patients’ ages or genders. Most complications (95%) occurred within 6 months following the joint replacement surgeries. Numbness and paresthesia due to nerve irritation of the superficial radial nerve (SRN) was the most prevalent complication (55 patients; 14%), with no significant association between surgical approach and SRN paresthesia (39 of 296 (13%) cases were performed using a dorsal approach, and 16 of 85 (19%) cases were performed using a radio palmar approach; *p*-value = 0.19). Most patients reported that numbness in the dorsal aspect of the first web space and thumb had resolved within a few weeks after surgery; however, one patient required surgical neurolysis of the nerve 6 months following their initial surgery because of continued dysesthesia. 

## 4. Discussion

In this study, we collected information regarding complications in thumb CMC joint replacement surgeries during the learning curve period in six major public hospitals, and we found that 6% of the surgeries had major complications and 25.4% had minor complications. A large majority of the complications occurred during the first 6 months after the procedure. 

Although a trapeziectomy and stabilization of the thumb metacarpal using different techniques is considered successful, multiple unsuccessful attempts have been made to find alternative procedures to enable faster recovery with less pain and increased strength. The complications and poor long-term survival rates of initial ball and socket implant designs deterred many surgeons from using them [12]. Modern designs have demonstrated significant improvements in implant survival rates, with more than 90% exceeding 10 years [13,14]. The concerns regarding peri-operative complications remain. Most reported complications could be related to technical errors, including dislocations, fractures, loosening, failures, or wear of the implants. Dumartinet-Gibaud et al. [15] noted that the number of complications due to surgeons’ technical errors was high during the first 30 procedures, but this number markedly decreased after that. Bricout [16] reported a failure rate of 7.7% in implementing the MAÏA™ prosthesis in a series of 156 patients. Although our study depicted the learning curve period of many surgeons, our failure rate was much lower, with only 2.9% of the cases resulting in trapeziectomies. 

A careful surgical technique is important for reducing the risk of implant failure, and it is especially crucial for a trapezial cup placement. Precise positioning is needed in order to avoid the excessive removal of bony circumferential support, the loss of initial press fit fixation, and trapezial fractures, which can lead to failures in osseointegration or even be an indication for an intra-operative change in plan to a trapeziectomy. Fracture of the trapezium may be treated by prolonged immobilization, conversion to cemented cup-fixation, or primary or secondary trapeziectomy [17]. In our series, fracture of the trapezium was the most common cause of prosthetic failure. Intra-operative fracture occurred in nine cases (2.4%), both with the MAÏA™ (5 cases) and the Touch^®^ implants (four cases), and in four of these cases, the prostheses were found to remain stable after several weeks of cast immobilization. In the other five cases, the patients were treated with trapeziectomies either immediately or early after their initial surgeries. A fracture of the trapezium may occur following trauma as well. In one case in our series, the patient fell on an outstretched hand while running, fractured the trapezium, and was treated with a trapeziectomy. Fractures of the metacarpus did not occur in our series.

**Figure 2:**
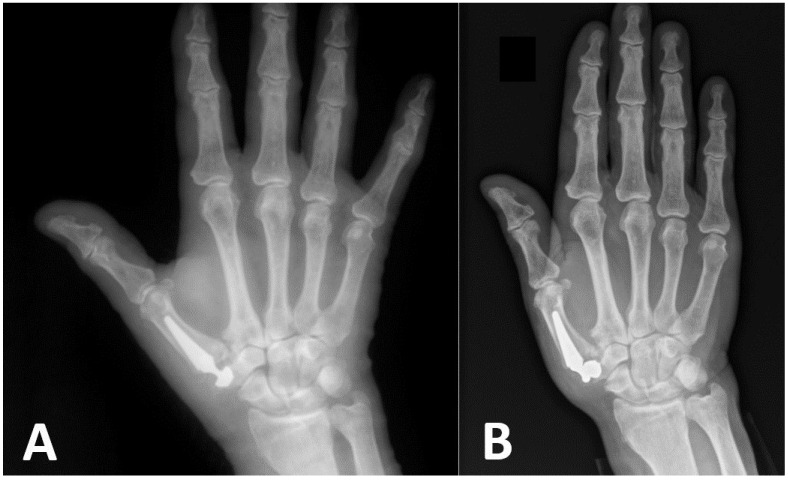
Immediate (**A**) and two-weeks’ (**B**) post-operative X-rays showing early cup loosening and dislocation of the prosthesis.

Implant loosening without a fracture may occur early following surgery because of an inability to achieve primary press fit fixation. Careful evaluation of radiographs to assess bone quality, the presence of cysts, and adequate trapezium height are essential steps in preoperative planning. Central cup placement [18], as well as preservation of the hard subchondral bone of the trapezium articular surface, may result in better mechanical fixation of the cup [19]. In four cases in our series, early loosening of an implant’s cup was diagnosed within several weeks following initial surgery, and all of them involved Touch^®^ prostheses. A typical case of such a complication is presented in Figure 2, where immediate post-operative X-rays demonstrated a cup positioned radially and dorsally to the center of the trapezium and early loosening of the cup with dislocation of the prosthesis 2 weeks post-surgery. The Touch^®^ implant has two cup designs. The conus cup is based on the concept that the axial forces maintain its press fit into the trapezium [14]. The hemispheric cup is similar to acetabular cups, and its force distributions may differ. Our results did not show any superiority of one design over the other, as two of the cups which failed were spherical and two were conical. In one case, deepening the cavity in the trapezium was sufficient for stabilizing the cup during revision surgery. In another case, a bone graft from the iliac crest was used to build the bone stock into which a cup was later implanted. In two cases, however, the surgeon was unable to stabilize a cup, and therefore, trapeziectomies were performed. We encountered one case of late loosening of the cup in our series, where the patient complained of new onset pain one year following hemispheric Touch^®^ JR surgery and underwent successful revision surgery with the insertion of a larger cup.

Dislocation rates reported in the literature are as high as 15%, and this may be a result of incorrect component position, bone impingement, soft tissue insufficiency, postoperative rehabilitation, and wear. Bricout [16] noticed that a prosthetic dislocation is the most common cause of early revisions and failures, and cup malposition was significantly associated with this risk. The least chance of dislocation was when a cup was positioned central and parallel to the proximal articular surface of the trapezium [20]. Bone impingement due to inadequate osteophyte removal may cause a cam effect and dislocation, and it should be recognized during surgery. Soft tissue insufficiency may also cause implant instability that may be overcome by using a longer neck or a dual-mobility implant.

Dislocations were a common complication in previous implant designs [21]. In our series, only two cases of dislocations occurred, and both were with a MAÏA™ semi-retentive implants. This was similar to recent reports of semi-retaining MAÏA™ prostheses [22], and it was lower compared to non-retaining MAÏA™ implants [23]. Andrzejewski [23] reported that more than half of the dislocations they studied occurred during the first postoperative week in patients immobilized with splints after surgery, and they suggested that postoperative protocol may affect early stabilization of the joint. Our post-operative regimen consisted of a bulky soft dressing without splint immobilization, and it might explain the low dislocation rates in our series. One dislocation was treated with revision of the implant neck to a longer size; however, this did not resolve the implant’s instability, and a decision was made during the procedure to remove the implant and perform a trapeziectomy. The dual-mobility design, originally introduced in total hip implants, combines a mobile polyethylene liner on a small metallic prosthetic head, articulating in a metallic cup. The polyethylene head increases the size of the head and, thus, reduces the risk of dislocation. In our series, no dislocations occurred in the double-mobility implants (neither the Touch^®^ nor the MAÏA™), and in one case, changing from a single- to a double-mobility implant was sufficient for stabilizing an unstable implant.

Mechanical failure of an implant is a theoretical risk in all implant designs. Intraprosthetic dislocations with detachment of the polyethylene cap from the metallic head have been described in two cases that used the Moovis dual-mobility implant [17]. One such event occurred in a Touch^®^ prosthesis in our series. Another case of intraprosthetic dislocation of the polyethylene component of a MAÏA™ single-mobility semi-retentive cup was diagnosed in the first follow-up visit, and it was successfully treated by re-inserting the polyethylene component into the metal cup in a revision surgery. To the best of our knowledge, there have been no previous reports of this complication.

Prosthetic joint infection (PJI) is a known and at times devastating complication in large joint arthroplasties. The known risk factors for PJI in large joints are obesity, diabetes mellitus, smoking, inflammatory arthropathies, and immunosuppressive medications [24]. There are few data regarding small-joint PJIs. In a systematic review by Remy et al., the deep infection rate of thumb CMC arthroplasties was estimated at 0.23% [25]. Superficial infections have been seldom reported. We encountered only one case of deep infections in our series, and it was an acute hematogenous infection according to Tsukayama’s classification of a total-knee PJI [26]. The infection occurred in a 63-year-old male without known risk factors 8 months following an uneventful JR surgery. The details of this case have been previously reported [27]. 

Most studies describing the results of JR surgeries have focused on functional outcomes and the suppression of pain; therefore, only major complications tend to be reported. Our study aimed to report the occurrence of all complications. This resulted in an overall high complication rate, as has been found in similar previous reports [16]. Several authors have reported De Quervain’s or other tenosynovitis as the most frequent minor complication, occurring in up to 21% of patients [16,28,29]. We also noticed that tendinopathies occurred at approximately the third month, when patients resumed their activities and used their hands more; however, the occurrence of this complication in our series was less than 5%.

Paraesthesia or dysesthesia in the SRN territory was the most common complication in our series, with a prevalence of 15%, occurring at a similar rate using the radio palmar and the dorsal approaches. This minor complication has been reported at lower rates of 2–3.8% [14,16] in previous studies. A possible explanation for the high reporting rate of SRN paraesthesia in our report was that the nature of the study focused on minor complications. It may also be related to the more prolonged or extensive approaches taken by the surgeons for which this technique is novel, as was the case in this study. 

There were several limitations to the study, including the variability in the follow-up periods. Similar to our report, previous studies have shown that more than 90% of complications occur within 6 months following JR surgeries [16]. Our follow-up period, however, was not long enough to assess long-term complications, especially loosening of the prostheses and their life spans. Another limitation of the study was that although we reported on a large cohort of patients, the number of complications was relatively low, with variations in the types of complications, and therefore, it was not possible to identify the risk factors for the various complications. Further studies with larger cohorts are needed to identify if patients’ or implants’ charecteristics influence failure rates in JR surgeries. The strength of the study was that it included multiple surgeons from multiple centers and it used two similar modern implants, as well as the surgeons’ learning curves for the new technique.

## 5. Conclusions

In conclusion, our study provides valuable insights into the complications associated with modern implant arthroplasties of the thumb CMC joint during the learning curve periods across six major public hospitals. In contrast to previous studies, our findings revealed a notably low 2.9% rate for short-term failures and other major complications. In cases necessitating revision surgeries, the viable option of a trapeziectomy, with or without ligament reconstruction or suspensionplasty, remain available. Predominant among the minor complications were instances of SRN irritation, which often resolved spontaneously, along with the occurrence of De Quervain tendinopathy.

Despite advancements in implant designs, concerns persist regarding peri-operative complications, with technical errors such as fractures and dislocations being noteworthy contributors. Our findings underscore the importance of careful surgical technique, especially regarding trapezial cup placement, to mitigate the risk of complications, with fracture of the trapezium being a notable concern in our series. Dislocation and other major complications in the modern implants were very low compared to previous reports. Thumb JR surgery requires expert skills, the learning curve is challenging, and there is a need for meticulous attention to detail during surgery, especially while placing the cup in a central location and in an adequate direction.

The main limitation of our study was the variable follow-up period, precluding a comprehensive assessment of long-term complications. Larger cohorts and extended follow-up durations are necessary to identify potential risk factors for specific complications. Despite these limitations, our study benefited from its inclusion of multiple surgeons across diverse centers, employing two similar modern implants and incorporating the learning curve aspect.

Future research should delve into refining surgical techniques to minimize complications, exploring novel implant designs, and conducting extensive, long-term studies to evaluate the durability of prosthetic joints. Addressing the limitations identified in our study will contribute to a more comprehensive understanding of the factors influencing the success and longevity of thumb CMC joint replacement surgery.

## Figures and Tables

**Table 1:** Complications in 381 patients from 2015 to 2022.

Major Complication—Implant Failure	N = 11,	2.9%
Intra-operative fracture of the trapezium, inability to stabilize cup	2	
Intra-operative fracture of the trapezium, cast immobilization, early loosening	3	
Fracture of the trapezium d/t fall 6 weeks post-operation, implant instability, trapeziectomy	1	
Early loosening without intra-operative fracture, trapeziectomy	1	
Dislocation, inability to stabilize following change to larger neck	1	
Prosthetic joint infection	1	
Unexplained continued pain	2	
**Major complications requiring surgical intervention**	N = 12,	3.1%
Dislocation treated with revision to dual-mobility implant	1	
Early loosening, re-stabilization of the cup	2	
Late loosening, revision to larger cup	1	
Intra-prosthetic dislocation	2	
Ruptured extensor pollicis longus	1	
Trapezium osteophytes and stiffness	1	
Superficial radial nerve neuritis, surgical neurolysis	1	
De Quervain’s tenosynovitis, surgical release	3	
**Minor complications**	N = 97,	25.4%
Intraoperative fracture of the trapezium, cast immobilization	4	
Superficial wound infection treated with PO antibiotics	7	
De Quervain’s tenosynovitis, conservative treatment	14	
Thumb or finger flexor tenosynovitis	6	
Superficial radial nerve paresthesia	57	
Carpal tunnel syndrome	8	
Palmar cutaneous branch median nerve, thumb digital paresthesia	6	
Complex regional pain syndrome	3	

## Data Availability

The data supporting the reported results can be obtained from the corresponding author.
